# Oral Endothelium Corneum Gigeriae Galli Therapy for Pancreatic Duct Stones: A Prospective Cohort Study

**DOI:** 10.5152/tjg.2022.22086

**Published:** 2022-12-01

**Authors:** Dujuan Cao, Huiyu Li, Jinxi Wang, Fang Zhang, Haoliang Zhao, Chongren Ren

**Affiliations:** 1 Department of Neurology, Shanxi Bethune Hospital, Shanxi Academy of Medical Sciences, Tongji Shanxi Hospital, Third Hospital of Shanxi Medical University, Taiyuan, China; 2 Department of General Surgery, Shanxi Bethune Hospital, Shanxi Academy of Medical Sciences, Tongji Shanxi Hospital, Third Hospital of Shanxi Medical University, Taiyuan, China

**Keywords:** Chinese medicine, chronic pancreatitis, endothelium corneum gigeriae galli, pancreatic duct stones, pancreatic stone protein

## Abstract

**Background:**

Pancreatic duct stones obstruct the pancreatic ducts and aggravate clinical symptoms of chronic pancreatitis. Only isolated case reports have shown that some drugs may be useful in dissolving pancreatic duct stones. Endothelium corneum gigeriae galli is a Chinese medicine widely used to cure multifarious lithiasis and maldigestion. This study aimed to evaluate the efficacy of endothelium corneum gigeriae galli oral therapy in the dissolution of stones and evaluate the improvement of clinical symptoms in patients with pancreatic duct stones.

**Methods:**

Sixty-eight patients with pancreatic duct stones were randomly divided into the endothelium corneum gigeriae galli and control groups. Endothelium corneum gigeriae galli was given orally to the endothelium corneum gigeriae galli group, and the placebo was given to the control group. Both groups were reviewed by computed tomography and magnetic resonance imaging; abdominal pain, exocrine and endocrine pancreatic function, and the nutritional status of patients were measured after the study.

**Results:**

The dissolution rate of the endothelium corneum gigeriae galli group was significantly higher than that of the control group (*P* = .002). The abdominal pain of the endothelium corneum gigeriae galli group was relieved more significantly compared to that of the control group (*P* < .001). The exocrine and endocrine pancreatic function of the endothelium corneum gigeriae galli group improved more significantly than that of the control group (*P* < .001). The nutritional status of the endothelium corneum gigeriae galli group was significantly higher than that of the control group (*P* = .003).

**Conclusion:**

Overall, oral endothelium corneum gigeriae galli treatment could dissolve pancreatic duct stones, relieve abdominal pain, improve exocrine and endocrine pancreatic functions, and control the deterioration of nutritional status. Endothelium corneum gigeriae galli treatment should be useful in pancreatic duct stones therapy.

**Main Points**

- Drug research and development for pancreatic duct stones (PDS) are increasing slowly in recent years, and therefore, endothelium corneum gigeriae galli (ECGG) should be an excellent treatment.
- Oral ECGG could dissolve PDS and relieve abdominal pain.
- Oral ECGG could improve exocrine and endocrine pancreatic functions in patients with PDS.

## Introduction

Chronic pancreatitis (CP) is a disease of multiple causes related to ongoing and non-reversible inflammation in the pancreas, leading to a loss of functional pancreatic tissue and both exocrine and endocrine deficiency.^1^ Pancreatic duct stones (PDS) are formed as a result of CP in about 50% of patients and may be situated in the Wirsung duct or lateral branches. Pancreatic duct stones cause or aggravate abdominal pain by blocking ductus pancreaticus, inducing upstream duct high tension, and exacerbating exocrine and endocrine pancreatic dysfunctions and poor nutritional status.^2^ Clearing these stones, easing high tension duct, soothing pain, and enhancing the quality of life are the primary objectives of treatment in patients with CP.

There are 3 treatment options for PDS, namely pharmacological, endoscopic, and surgical drainage. Endoscopic and surgical therapies have drastically improved in recent years.^2-4^ However, the development of pharmacological drainage has been slow due to the lack of effective therapies and adverse effects. Only isolated case reports have described some drugs, such as citrate and trimethadione, to be potentially useful in dissolving PDS.^5,6^

Chinese medicine has been widely used in clinical settings for thousands of years. Endothelium corneum gigeriae galli (ECGG), made by gizzard lining after drying, is described in the Chinese pharmacopeia-2015 as a traditional Chinese medicine usually used to cure multifarious lithiasis and maldigestion. Endothelium corneum gigeriae galli is edible and has no overt side effects.^7,8^ Therefore, it may be an excellent treatment for PDS.

Given all this, our study primarily aimed to test the efficacy of the dissolution of stones and pain relief, improvement of exocrine and endocrine pancreatic function, and variation of nutritional status after ECGG oral therapy in patients with PDS in the General Surgery of the Shanxi Bethune Hospital.

## Materials and Methods

This prospective cohort study with a 24-month follow-up period collected data on patients with PDS who presented to the General Surgery Clinic of the Shanxi Bethune Hospital between January 2018 and December 2020. All patients were evaluated by computed tomography (CT, 64-slice spiral Siemens, Berlin, Germany) and magnetic resonance cholangiopancreatography (MRCP) using magnetic resonance imaging (MRI, 1.5-T Siemens, Berlin, Germany) according to the IAP/APA/JPS/EPC guidelines for the diagnostic cross-sectional imaging and severity scoring of chronic pancreatitis-2018 and M-ANNHEIM diagnostic criteria.^9,10^ Inclusion criteria were a maximum diameter of PDS > 0.3 cm; reluctance to treatment with endoscopic and surgical drainage; failure of endoscopic drainage and reluctance to treatment with surgery; and contraindications to surgical and endoscopic treatment, including cardiopulmonary insufficiency, uremia, hepatic failure, and other conditions. Exclusion criteria were poor self-control in daily life, including the inability to abstain from alcohol and smoking and maintain a diet; pregnancy; subacute exacerbation of CP; diabetes mellitus type 2; hyperparathyroidism; mass-CP of pancreatic head >4 cm; age < 18 or > 80 years; severe portal hypertension; Billroth II gastrectomy; other complications of pancreatitis that needed surgery; segment pancreatectomy; potential pancreatic carcinoma; and expectation of life <2 years.

The study was approved by the ethics committee of the Shanxi Medical University. After being informed of the potential risks of the study, consent was signed by all participants. The main clinical features before starting treatment were recorded. Patients with PDS were randomly divided into the ECGG and control (CON) groups.

The raw ECGG was purchased from Beijing Tong Ren Tang Co., Ltd. (Beijing, China) and examined by 2 professors at the Shanxi University of Traditional Chinese medicine. Endothelium corneum gigeriae galli was mixed into food in powder form and was given orally at a dose of 2 g 3 times daily. The same dose of placebo was given to the CON group. Both groups were under the routine treatment of CP, including the cessation of alcohol consumption and smoking, a low-fat diet, pain relief, pancreatin supplementation, and diabetes control.

In this study, the nutritional status of patients was based on body mass index (BMI); abdominal pain was either constant or episodic and substantially variable based on the visual analog score (VAS); exocrine pancreatic function was investigated using the fecal elastase-1 test (FE-1); endocrine pancreatic function was assessed using fasting blood glucose (FBG), oral glucose tolerance test (OGTT), and acute insulin release (AIR) tests.

Patients in both groups were reviewed every 6 months by color ultrasonography and every 12 months by CT and MRI. The results of CT and MRI were further verified by 2 specialists. Body mass index, VAS, FE-1, FBG, OGTT, and AIR were measured in both groups in the post-study period. Stool samples were collected and sent to the laboratory of the Shanxi Bethune Hospital for FE-1 assessment. Fecal elastase-1 level was determined with an enzyme-linked immunosorbent assay (ELISA) kit (ScheBo Biotech Ag, Giessen, Germany). Fasting blood glucose was measured using venous blood collected in the early morning. The blood glucose 2 hours after taking 75 g of oral glucose was measured in venous blood. For AIR, 4 mL of fasting venous blood was collected from cubital vein punctures with the patients in the supine position; 20 mL of 25% arginine was injected (injection time was within 30 seconds) (Shanghai Xinyi Jinzhu Pharmaceutical Co., Ltd. Shanghai, China); 2 mL of venous blood was collected at 2, 4, 6, and 8 minutes after injection; blood samples were sent to the laboratory of the Shanxi Bethune Hospital, and C-peptide levels were measured while fasting and at 2, 4, 6, and 8 minutes (A1, A2, A3, A4, and A5). Acute insulin release was calculated as follows: AIR = (A2+A3+A4+A5)/4 − A1.

The adverse side effects of ECGG were monitored in both groups of patients every 2 months. Additionally, a blood examination was performed to check the toxicity of ECGG to the blood, liver, and kidney.

### Statistical Analysis

The dissolution rate of PDS was assessed using the chi-square test. Quantitative data consistent with normal distribution were shown as mean ± standard deviation (SD) and evaluated using Student’s *t*-test. For variables with abnormal distribution, data were shown as median ± quartile range and evaluated using the Kruskal–Wallis test using Statistical Package for the Social Sciences version 22.0 (IBM, Armonk, NY, USA). *P* <.05 was considered statistically significant.

## Results

### Clinical Characteristics of Patients with Pancreatic Duct Stones

Totally 103 patients matched condition, 38 unsigned their consent (n = 34) or did not have self-control (n = 4), and 7 patients were excluded due to subacute exacerbation of CP. Finally, 68 patients were included. The main clinical characteristics prior to treatment are shown in Table 1. The median age was 41.23 ± 15.71 (range, 25-63) years, and the BMI was 21.44 ± 1.73 (range, 15-26) kg/m^2^. Males accounted for an overwhelming 83.82% of patients. The average course of illness took 4.67 ± 2.69 years. Alcohol abuse (alcohol consumption > 80 g/day) was the most common etiological agent found in 75.0% of patients. Smoking and other reasons are some of the known etiological agents found in approximately 69.12% and 13.24% of patients, respectively. All patients complained of abdominal pain. Persistent weight loss was found in 92.65% of patients; 35.29% of patients were diagnosed clinically with diabetes according to the standards of the American Diabetes Association-2018.^11^ Some patients (17.65%) presented with severe pancreatic exocrine insufficiency manifestations, such as fatty diarrhea. According to the widely used M-ANNHEIM diagnostic criteria of CP, 39 patients were classified as stage II and 29 patients were stage III.

Dissolution of Pancreatic Duct Stones by Endothelium Corneum Gigeriae Galli Treatment

**Figure 1.**
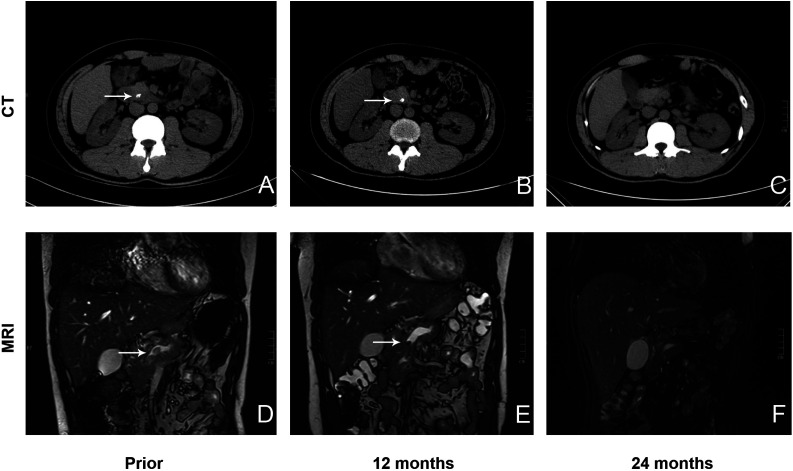
Dissolution of PDS with oral ECGG in a patient who had previously undergone ERCP and pancreatic sphincterotomy followed by balloon basket. (A) Cross-section CT image of the pancreas directly before treatment showing a relatively large stone in the head of the pancreas (arrows). (B) After oral ECGG treatment for 12 months, CT cross-section image showed a reduction in the size of PDS (arrows). (C) After oral ECGG for 24 months, CT cross-section image showed complete dissolution of PDS. (D) Magnetic resonance imaging coronal images of the pancreas directly before treatment showed a 0.9 cm diameter stone and many small stones in the pancreas head. (E) After oral ECGG treatment for 12 months, MRI coronal images showed a reduction in size and number of stones (arrows). (F) After oral ECGG treatment for 24 months, MRI coronal images showed complete dissolution of PDS. PDS, pancreatic duct stones; ECGG, endothelium corneum gigeriae galli; CT, computed tomography; MRI, magnetic resonance imaging; ERCP, endoscopic retrograde cholangiopancreatography.

A 43-year-old man with CP for 5 years was diagnosed with PDS, and he had a history of alcohol abuse. He underwent the standard operating procedure of endoscopic retrograde cholangiopancreatography (ERCP) and pancreatic sphincterotomy followed by a balloon basket for the stone embedded in the head of the pancreas in the Wirsung duct. The result was unsatisfactory. Because he was reluctant to undergo surgical treatment, he was recommended to participate in our study. Computed tomography and magnetic resonance imaging were used to evaluate the results of ECGG treatment every 12 months. Computed tomography and magnetic resonance imaging of the abdomen directly before treatment showed a 0.9 cm diameter stone and many small stones in the pancreas head (Figure 1A, D). A significant decrease in dimensions and numbers of stones after 12 months of treatment was observed (Figure 1B, E), and the stones disappeared after 24 months of treatment (Figure 1C, F).

Therapeutic Effect of Endothelium Corneum Gigeriae Galli on Pancreatic Duct Stones The result of ECGG treatment in the dissolution of PDS was monitored by CT and MRI (effective or none). Effective treatment was defined as the disappearance of PDS in the CT and MRI scan; none was considered when there was a remnant stone in the pancreatic duct. The ECGG group included 35 patients, and the CON group included 33 patients by the end of follow-up. The effect of ECGG treatment on PDS during the 24 months of follow-up is shown in Table 2. The dissolution rate of the ECGG group was 54.29% (19/35) and was significantly higher than that of the CON group (18.18%) (6/33) (*P* = .002).

### Endothelium Corneum Gigeriae Galli Relieved Abdominal Pain and Improved Exocrine and Endocrine Pancreatic Functions and Nutritional Status

The values of VAS, FE-1, FBG, OGTT, and AIR of 68 patients pre- and post-treatment during the 24 months of follow-up are shown in Table 3.

**Figure 2.**
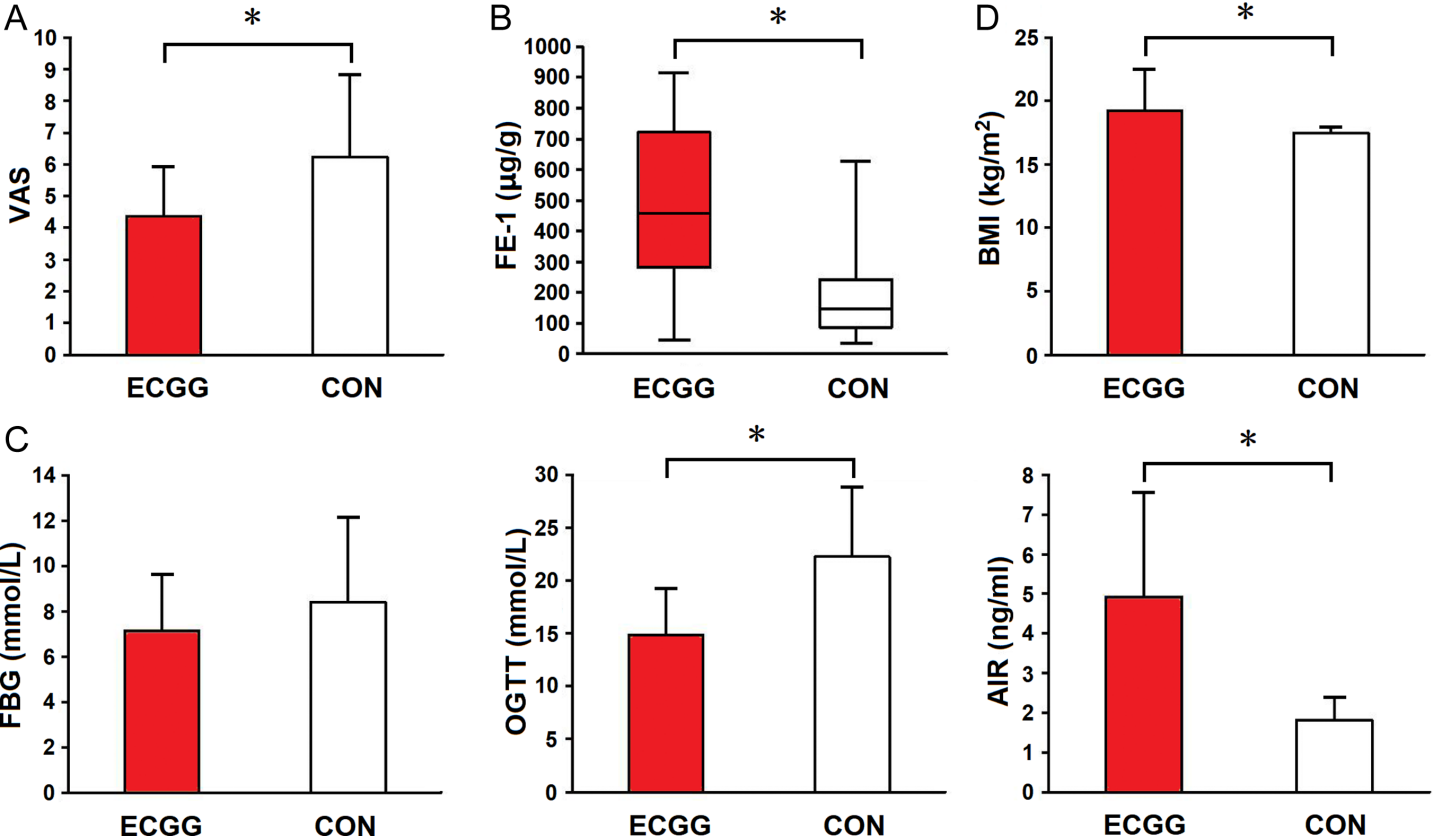
Endothelium corneum gigeriae galli relieved abdominal pain and improved exocrine and endocrine pancreatic function and nutritional status. (A) Abdominal pain detected by VAS. Data were shown as the mean ± SD of both groups; ^*^ *P* < .05, Student’s *t*-test. (B) Exocrine pancreatic function was determined by FE-1. Data were shown as the median ± QR of both groups; ^*^ *P* < .05, Kruskal–Wallis test. (C) Endocrine pancreatic function was evaluated by FBG, OGTT, and AIR. Data were shown as the mean ± SD of both groups. ^*^ *P* <.05, Student’s *t*-test. (D) Nutritional status was detected by BMI. Data were shown as the mean ± SD of both groups; ^*^ *P* < .05, Student’s *t*-test. CON, group control; VAS, visual analog scale; SD, standard deviation; FE-1, fecal elastase-1; FBG, fasting blood glucose; OGTT, oral glucose tolerance test; ECGG, endothelium corneum gigeriae galli; QA, quartile range.

Abdominal pain is the dominantly clinical characteristic of PDS, and with the dissolution of PDS, abdominal pain was alleviated to some degree. Abdominal pain was assessed by VAS at the end of the follow-up. The VAS of the ECGG group was 4.37 ± 1.55, whereas that of the CON group was 6.24 ± 2.57. The VAS of the ECGG group declined more significantly compared to that of the CON group (*P* < .001; Figure 2A).

There are many ways to diagnose pancreatic exocrine insufficiency. The FE-1 estimation is a credible and affordable test to detect pancreatic exocrine function.^12^ Pancreatic exocrine function was investigated by FE-1 at the terminal follow-up. The FE-1 of the ECGG group was 454 (280.5, 721) μg/g and significantly higher than that of the CON group, which was 155 (87.5, 235) μg/g (*P* < .001; Figure 2B).

The gold standard for the diagnosis of pancreatic endocrine insufficiency is FBG, OGTT, and AIR.^13^ The arginine stimulation test can be used to avoid symptoms of impaired insulin secretion, which will affect testing with glucose. The pancreatic endocrine function was evaluated by FBG, OGTT, and AIR at the end of the follow-up. Although there was no significant difference in FBG between the ECGG and CON groups (7.15 ± 2.57 mmol/L vs 8.41 ± 3.77 mmol/L, *P* = .109; Figure 2C), the blood glucose at 2 hours of OGTT in the ECGG group was 14.84 ± 4.44 mmol/L and was significantly lower than that of the CON group, which was 22.25 ± 6.71 mmol/L (*P* < .001; Figure 2C). The patterns of AIR were similar to those of OGTT. The AIR of the ECGG group was 4.91 ± 2.65 ng/mL and was significantly higher than that of the CON group, which was 1.80 ± 0.59 ng/mL (*P* < .001; Figure 2C).

By the end of the 24-month follow-up period, the BMI of the ECGG group was 19.25 ± 3.25 (range, 13-25) kg/m^2^ and was significantly higher than that of the CON group, which was 17.21 ± 0.40 (range, 16-19) kg/m^2^ (*P* = .003; Figure 2D.).

### Side Effect of Endothelium Corneum Gigeriae Galli during Follow-up

Seven patients (20.0%) experienced constipation in the ECGG group, which improved after oral lactulose. Two patients (5.71%) complained of mild skin allergy, which improved after oral diphenhydramine. No laboratory findings suggesting ECGG toxicity were noted in the blood, liver, and kidney in both groups during the 24 months of follow-up.

## Discussion

Pancreatic duct stones are formed as a result of CP and may be found in Wirsung ducts or the lateral branches. Pancreatic duct stones obstruct the pancreatic ducts and produce duct hypertension leading to pain and the typical feature of CP. The major risk factors for PDS include alcoholism, smoking, metabolic disturbances, environmental conditions, and anatomical abnormalities.^2^ Our results are consistent with these findings (Table 1). Although cholelithiasis is the primary risk for acute pancreatitis,^14,15^ there was no definitive result in our study (Table 1).

Pancreatic stone protein (PSP) might act as a suppressant in PDS formation. Multiple causal factors, such as alcohol abuse, smoking, and pancreatic injury, led to a cut in PSP production. Pancreatic stone protein decrease contributes to the over-saturation of calcium carbonate in the succus pancreaticus. This calcium carbonate is then deposited over an inner nidus to form the PDS.^16^ Therefore, regardless of the etiology and pathogenesis of CP, the structure and composition of PDS are basically identical. Therefore, we theorized that restoring the PSP secretory function of the pancreas may induce the dissolution of PDS.

Endothelium corneum gigeriae galli is the internal wall of the sand-sac of the internal stomach membrane of the domestic chicken. Endothelium corneum gigeriae galli contains complex chemical components and many pharmacological effects. The contents of ECGG mainly include proteins such as pepsin, amylase, keratin and polysaccharides such as liminose, glucose, trehalose, mannose, and galactose. The proteins of ECGG could promote gastrointestinal peristalsis, regulate digestive enzyme secretion, and alleviate the fibrosis of the pancreas, therefore relieving the painfully swollen abdomen, expelling PDS, inhibiting the formation of PDS, and so on.^8^ The polysaccharides in ECGG, such as rhamnose, glucose, fucose, mannose, and galactose, might induce the dissolution of calcium carbonate.^7,8^ Hence, ECGG may be the best candidate for the treatment of PDS.

In this study, we found that the PDS dissolution rate of the CON group was 18.18% by ordinary treatment for CP (Table 2). Pancreatic duct stone autolysis in the natural history of CP has been recorded in some cases.^17^ It was reported that approximately 30% of CP cases presented a marked reduction of calcium deposition in the later stages of CP, and PDS autolysis is a frequent pathological process.^17,18^ The reason behind this may be that drinking and smoking cessation partially restore the secretory functions of the pancreas. The dissolution of PDS in this research is mainly owed to the effect of ECGG treatment because the dissolution rate of the ECGG group was significantly higher than that of the CON group (*P *= .002; Table 2). One possible explanation for this result is that ECGG promotes PSP secretion and induces the dissolution of calcium carbonate.

Abdominal pain is one of the major manifestations of PDS, with a survey of the incidence of 50%-90%.^1,2^ Pancreatic duct stones block the pancreatic duct and increase duct pressure which aggravates abdominal pain. Severe abdominal pain often results in the use of acesodyne.^19^ In this research, all patients showed varying degrees of abdominal pain (Table 1). With the ECGG treatment, abdominal pain was alleviated to some degree. The VAS of the ECGG group was significantly lower than the CON group (*P *< .001; Figure 2A). The main reason behind this phenomenon is the dissolution of the PDS. Unfortunately, the symptoms did not completely disappear with PDS dissolution. The etiology of the abdominal pain in CP is extremely complicated. Chronic pancreatitis inflammation is commonly correlated with the release of inflammatory cytokine from wounded cells, stimulating mast cells, basophilic granulocyte, platelets, and so on, leading to the transmission of pain signals to the central nervous system.^1,19^

Pancreatic exocrine function deficiency is a critical reason for maldigestion and is a common symptom of CP. Elastase-1 is discovered in pancreatic juice and is not degraded while transmitting through the alimentary canal.^13^ The concentration of elastase-1 is enriched 6 times in feces and can be detected using an ELISA kit. Early documents of FE-1 demonstrated a good correlation with the outcomes of pancreatic exocrine function.^13,20^ Morphological change in CP is also relevant to low FE-1 concentration, such as ERCP and MRCP.^21^ The threshold of FE-1 was set to 200 μg/g, and the diagnostic sensitivity and specificity of CP were 66.4% and 95.7%, respectively.^13,21^ After the treatment, the pancreatic exocrine function of the ECGG group was significantly higher than that of the CON group (*P *< .001; Figure 2B). This may result from PDS dissolution and ECGG stimulating the secretion of several digestive enzymes in the pancreas.

Pancreatic endocrine function deficiency of CP is known to induce type 3c diabetes mellitus (T3cDM).^22^ Type 3c diabetes mellitus is also one of the major manifestations of CP. With ECGG treatment, OGTT and AIR were improved significantly (*P* < .001; Figure 2C). This may be explained by the fact that PDS dissolution contributed to the recovery of pancreatic endocrine function and that ECGG stimulates insulin secretion.^7^ However, there was no significant difference in FBG between the ECGG and CON groups (*P* = .109 ; Figure 2C). Of note, T3cDM has its features. Although there are a great number of apoptotic β cells in CP, the secretory capacity of remnant β cells is preserved. Typical evidence is that ketoacidosis used to be a rare occurrence in T3cDM compared to other types of diabetes mellitus.^22,23^ The above-mentioned finding was validated by our study.

Persistent weight loss is another typical symptom of CP. It is a result of the concerted action of multi-factors such as pain, maldigestion, and diabetes. The BMI of the ECGG group was significantly higher than that of the CON group (*P* = .003; Figure 2D). Deterioration of nutritional status was controlled by oral ECGG. This result may be correlated with soothed pain, ameliorated appetite, and well-controlled diabetes.

However, this research has certain limitations. All patients were enrolled from a single-center institution which partially led to the problem of selection bias. Moreover, the plasma concentration of ECGG should not be detected, which affects the experimental results to some extent. Finally, the exact mechanism of ECGG therapy for PDS requires further verification.

In conclusion, our study supports the use of ECGG treatment for the dissolution of PDS in patients with CP, which showed relieved abdominal pain, improved exocrine and endocrine pancreatic functions, and controlled deterioration of nutritional status. In the future, PDS dissolution by oral ECGG could be combined with endoscopic and surgical therapy to achieve better CP outcomes.

## Figures and Tables

**Table 1.** Clinical Characteristics of Patients with PDS

	Total Population (n = 68)
Age, mean ± SD (years)	41.23 ± 15.71
BMI (kg/m^2^), mean ± SD	21.44 ± 1.73
Male (%)	57 (83.82)
Average course, mean ± SD (years)	4.67 ± 2.69
**Etiology (%)**	
Alcohol abuse	51 (75.00)
Smoking	47 (69.12)
Other reasons	9 (13.24)
**Symptoms (%)**	
Abdominal pain	68 (100.0)
Weight loss	63 (92.65)
Diabetes	24 (35.29)
Fatty diarrhea	12 (17.65)

SD, standard deviation; BMI, body mass index; PDS, pancreatic duct stones.

**Table 2.** Effect of ECGG Treatment on Dissolution of PDS

**Group**	**Effective**	**None**	**Total Population**	**Dissolution Rate (%)**	***P*** ^*^
**ECGG**	19	16	35	54.29	.002
**Control**	6	26	33	18.18	

^*^Based on the chi-square test.

ECGG, endothelium corneum gigeriae galli.

**Table 3.** Effect of ECGG Improvement in Clinical Symptoms in Patients with PDS

Group	VAS	FE-1^*^ (μg/g)	FBG (mmol/L)	OGTT (mmol/L)	AIR (ng/mL)	BMI (kg/m^2^)
PRE (n = 68)	6.56 ± 2.35	160.3 ± 125.05	7.98 ± 3.02	21.32 ± 6.12	2.13 ± 1.12	17.52 ± 1.38
POST						
ECGG (n = 35)	4.37 ± 1.55	454 ± 220.30	7.15 ± 2.57	14.84 ± 4.44	4.91 ± 2.65	19.25 ± 3.25
	CON (n = 33)	6.24 ± 2.57	155 ± 73.75	8.41 ± 3.77	22.25 ± 6.71	1.80 ± 0.59	17.21 ± 0.40

^*^Abnormal distribution, data were shown as median ± QR.

PRE, pre-treatment; POST, post-treatment; QA, quartile range; VAS, visual analog scale; FE-1, fecal elastase-1; FBG, fasting blood glucose; OGTT, oral glucose tolerance test; AIR, acute insulin release; BMI, body mass index; CON, control; ECGG, endothelium corneum gigeriae galli; PDS, pancreatic duct stones.
